# Hematopoietic Stem Cells and the Immune System in Development and Aging

**DOI:** 10.3390/ijms24065862

**Published:** 2023-03-20

**Authors:** Daniil Shevyrev, Valeriy Tereshchenko, Tatiana N. Berezina, Stanislav Rybtsov

**Affiliations:** 1 Centre for Cell Technology and Immunology, Sirius University of Science and Technology, Sirius, 354340 Sochi, Russia; dr.daniil25@mail.ru (D.S.);; 2 Department of Scientific Basis of Extreme Psychology, Moscow State University of Psychology and Education, 127051 Moscow, Russia; 3 Centre for Regenerative Medicine, University of Edinburgh, Edinburgh EH8 9YL, UK

**Keywords:** aging, aging biomarkers, immune aging, hematopoietic stem cells, HSC, pre-HSC, HSC precursors, inflammation, immune senescence, haematopoietic hierarchy, haematopoietic clonality

## Abstract

Hematopoietic stem cells (HSCs) support haematopoiesis throughout life and give rise to the whole variety of cells of the immune system. Developing in the early embryo, passing through the precursor stage, and maturing into the first HSCs, they undergo a fairly large number of divisions while maintaining a high regenerative potential due to high repair activity. This potential is greatly reduced in adult HSCs. They go into a state of dormancy and anaerobic metabolism to maintain their stemness throughout life. However, with age, changes occur in the pool of HSCs that negatively affect haematopoiesis and the effectiveness of immunity. Niche aging and accumulation of mutations with age reduces the ability of HSCs to self-renew and changes their differentiation potential. This is accompanied by a decrease in clonal diversity and a disturbance of lymphopoiesis (decrease in the formation of naive T- and B-cells) and the predominance of myeloid haematopoiesis. Aging also affects mature cells, regardless of HSC, therefore, phagocytic activity and the intensity of the oxidative burst decrease, and the efficiency of processing and presentation of antigens by myeloid cells is impaired. Aging cells of innate and adaptive immunity produce factors that form a chronic inflammatory background. All these processes have a serious negative impact on the protective properties of the immune system, increasing inflammation, the risk of developing autoimmune, oncological, and cardiovascular diseases with age. Understanding the mechanisms of reducing the regenerative potential in a comparative analysis of embryonic and aging HSCs, the features of inflammatory aging will allow us to get closer to deciphering the programs for the development, aging, regeneration and rejuvenation of HSCs and the immune system.

## 1. Introduction

Hematopoietic stem cells (HSCs) are the basis for maintaining the entire hematopoietic system of the organism throughout life [1]. In adults, they are in a dormant state and periodically enter into an asymmetric division, giving rise to an array of multipotent and unipotent precursors that are involved in the renewal of specialized blood cells and the immune system [2]. The pool of HSCs is successfully replenished by symmetrical division (self-renewal), and therefore, only a small part of these cells is spent during life [3]. However, when aging along with the body interacting with bone marrow, HSCs gradually lose their ability to maintain the entire hematopoiesis [4].

Increasing age-related insufficiency of DNA repair leads to the accumulation of mutations accompanied by epigenetic, transcriptomic, and proteomic changes, metabolic dysregulation, and disruption of HSC differentiation programs [5,6,7,8]. Degeneration of the bone marrow and stem cell compartment shifts the quantitative ratio among hematopoietic stem and blood progenitor cells (HSPCs), which, together with the involution of the thymus, contributes to an imbalance between lymphopoiesis and myelopoiesis [9,10,11,12]. Concurrently, the mutation load, which is transmitted from HSCs to daughter cells, negatively affects the functional activity of various populations of specialized blood cells [13,14]. In this review, we have summarized the current understanding of the development and life of HSCs, as well as the mechanisms of aging of HSCs in the context of a negative impact on the function of the immune system.

In this review, particular attention is paid to the ontogeny of hematopoietic stem cells (HSCs) from their early specialization to old age. The review also highlights the existing contradictions and discussions in the area of HSC research, as well as the similarities and differences in HSCs at different stages of ontogeny. Given this focus, we do not discuss in detail other specialized aspects of hematopoietic development. We invite readers to refer to other recent, excellent reviews on specialized topics on HSC hematopoietic hierarchy, function, physiology, and aging [15,16,17,18,19,20,21,22,23,24,25,26,27,28,29,30,31].

## 2. HSC Juvenescence

According to recent publications, the earliest HSC precursors capable of maturing into fully functional blood stem cells appear in mice from the embryonic days 8–9 during remodeling of the dorsal aorta in an area called the para-aortic splanchnopleure. This occurs after the onset of the heartbeat [32]. 

Further, all HSC maturation events occur in the region of the dorsal aorta, gonads, and mesonephros (AGM). Apparently, at the time of remodeling, when the aortic lumen begins to form under the influence of shear stress [33,34], presumably from PDGFRα+ mesodermal progenitor [35] via VE-cadherin+ (VC+) haematogenic endothelium (HE) [36,37,38], pro-HSC appeared (VC+CD41^lo^CD43-CD45-Lin-)—the first ancestor committed to HSC [39,40].

Single cell analysis predicted the existence of an intermediate precursor of HE, which is apparently multipotent and produces several cell types including endothelial cells, hematopoietic cells, for the needs of developing embryos, and for adult haematopoiesis As shown, HE is characterized by accessibility of chromatin enriched for SOX, FOX, GATA, and SMAD motifs, as well as the expression of HSC precursor specification factor Runx1 [38]. New-born mice transplantation studies have shown that intra-embryonic HE without a heart beat is capable of producing AA4.1+CD19+B220^lo-neg^ precursors of innate B1 cell and marginal zone B cells [41], lymphoid tissue inducer cells [42] and precursors of embryonic T lymphocytes [43], and other cells also derived from the HE of yolk sac [21,37]. However, the first progenitor capable of maturing into fully functional HSCs in ex vivo culture is the pro-HSC. In addition, pro-HSCs can differentiate into type I pre-HSCs. (VC+CD41^lo^CD43+CD201^hi^CD45-Lin-) and sequentially in type II pre-HSC with the phenotype VC+CD41^lo^CD43+CD201+CD45+Lin-. Note, the latter is the immediate precursor to maturation into definitive HSCs (dHSCs) [39,44,45]. Fully matured dHSCs in AGM are already able to give rise to all blood lines and provide complete adult haematopoiesis, without prior maturation into immunodeficient new-born mice or in ex vivo culture [46]. Late progenitors of dHSCs can be partially detected when transplanted into new-born immunodeficient mice after myeloablation with busulfan [47]. Note, the special culture of maturation ex vivo at the liquid–air interface with high efficiency can produce definitive HSCs from the earliest (E9) precursors, capable of repopulating immunocompetent adult wild-type animals after myeloablation. In such experiments, the total number of HSC precursors at different stages of development, their early hierarchy, the main signaling pathways, and the most important growth factors necessary for their expansion and differentiation were determined [39,44,45,48,49,50,51,52,53].

Prior final maturation, at the end of day 9 of development, mouse embryonic HSC precursors begin to proliferate when their pool increases from a few single cells to ~100 pre-HSCs. Interestingly, such amplification of the pre-HSC pool takes only 48 h [48]. That pre-HSCs divide at a faster rate, going through at least 3–4 divisions in 48 h, before maturation and migration to the fetal liver [48,54].

### 2.1. First Definitive HSCs

The first hematopoietic stem cells in embryogenesis that can restore all hematopoietic lineages and creating colonies of multipotent cells in the spleen (CFU-S) when transplanted into sublethally irradiated animals are called definitive HSCs (dHSCs) [46,55,56]. They appear in the AGM region on the 11th day of embryogenesis. In humans, the first blood stem cells arise in Carnegie Stages (CS) 14–15 on days 32–36 of the embryo development, also in the AGM region [57,58,59]. As in mice, the first human HSCs appear in the ventral aspect of the aorta in emerging intra-arterial hematopoietic clusters. (IAHC) [60,61,62]. Thus, in the embryo, the primary niche for maturation and reproduction of HSCs is the ventral aortic endothelium and IAHC on the ventral aspect of dorsal aorta. In addition to the aortic endothelium, several types of cells are involved in the formation of such a niche. Macrophages with an inflammatory signature in the IAHC are involved in the maintenance and development of HSC [63]. GATA3 mediated production of catecholamines by the sympathetic nervous system in ventral mesenchyme of dorsal aorta contributes to the development of HSCs [64]. The key role of niche secreted factors such as IL3, Kit ligand, BMP inhibitors in the expansion of HSC progenitors has also been demonstrated [39,44,51,52,65].

### 2.2. High Regenerative Potential of Embryonic HSCs

**Figure 1:**
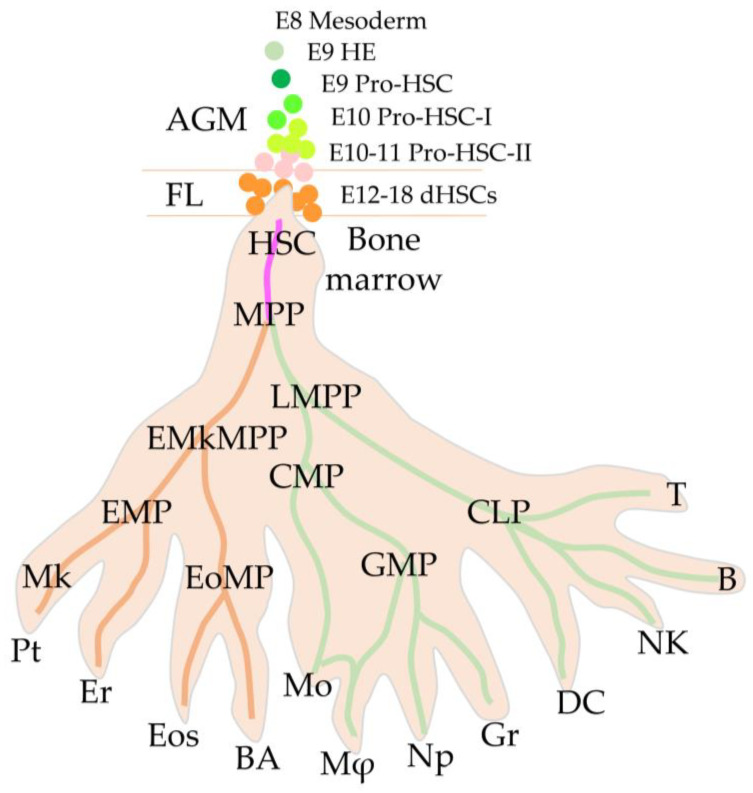
Development of blood stem cells from embryonic progenitors in AGM and fetal liver, to adult hematopoietic hierarchy in bone marrow. AGM—aorta-gonad-mesonephros; FL—fetal liver, HE—hematogenic endothelium, HSC—hematopoietic stem cell; MPP—multipotent progenitor; LMPP—lymphoid-primed multipotent progenitor; CMP—common myeloid progenitor; EMkMPP—erythroid-megakaryocyte-primed multipotent progenitor; EoMP—eosinophil-mast cell progenitor; EMP—erythro-myeloid progenitor; GMP—granulocyte-monocyte progenitor; CLP—common lymphoid progenitor; Mk—megakaryocyte; Pt—platelet; Er—erythrocyte; Eos—eosinophils; BA—basophils; Mo—monocyte; Mφ—macrophage; Gr—granulocyte; Np, neutrophils; DC—dendritic Cell; NK—natural killer; B-cell and T-cell. The brown line shows the GATA1 dependent and the green GATA1 independent hierarchy. The pink line shows the area of development of multipotent progenitors. Hematopoietic hierarchy inspired by [15] data and updated according to [40,67] as well as other data referenced in the text.

At CS 17–18 of maturation in humans and on day 12 in mice, HSC precursors in the AGM region acquire the ability to migrate and homing. Thus, the “childhood” period of development of HSCs ends, and they enter “adult life” [40,66]. These young AGM-dHSCs are fully prepared to perform the basic functions of maintaining the entire hierarchy of the hematopoietic system (Figure 1).

Surprisingly, in humans at the stage of CS 15–18, dHSCs have an extraordinary regenerative potential. They can produce numerous offspring, repopulate recipients at a high level, and give rise to all blood cell lines. Thus, it has recently been shown that when AGM-derived dHSC is transplanted into immunodeficient mice, it is able to produce 600–1600 fully functional daughter HSCs [68]. Note, that transplantation of HSCs from umbilical cord blood (UCB) gives no more than three daughter HSCs. Also, HSCs derived from adult bone marrow appear to have even less self-renew ability and less regenerative potential compared to embryonic HSCs and UCB-HSCs [68,69,70,71]. Note, the efficiency of the recipient’s repopulation depends on the number of transplanted HSCs and on the homing activity of the transplanted HSCs [72].

### 2.3. Migration to the Fetal Liver through the Placental Labyrinths

Perhaps, pre-HSC migration to the fetal liver begins before the full maturation of definitive HSCs. That is why the sudden appearance of a large number of maturing HSCs, first in the placenta, and then fully functional HSCs in the fetal liver, reflects the pilgrimage of these cells through the circulation. At the time of migration, such cells can be found even in the vessels of the head, and possibly in the lungs of the embryo before fetal liver homing [73]. Apparently, the main route of their youthful journey passes along the main arteries [74] and labyrinths of the placenta [75,76]. Perhaps they undergo final maturation in the placenta to eventually migrate to the fetal liver. It is possible that in humans, the placenta serves as an additional niche supporting HSC and haematopoiesis until birth [77].

However, the fetal liver is the main site of propagation and accumulation of HSCs before birth. In mice, colonization of the fetal liver by dHSCs begins on day 12 of embryonic development, whereas in humans it begins at 18 CS (44 days post-fertilization) [62,68,78].

HSCs go through the stages of maturation, migrating through the vascular labyrinths in the placenta, after which they enter the fetal liver, where the cell cycle rate is reduced [54]. Mouse HSCs in the fetal liver undergo at least four divisions after initial colonization and gradually form the final pool of HSCs ready to migrate to the bone marrow over first days after birth [66,79,80]. It is worth noting that the liver of a new-born mouse retains its hematopoietic activity for two weeks after birth [80,81,82]. This allows all fetal liver HSCs to migrate through new-born blood circulation to the bone marrow and enter a dormant state [80]. Thus, the regenerative potential of HSCs decreases already in the umbilical cord blood of a new-born human [68] and continues to further decline in HSCs from the bone marrow and with subsequent aging [83,84].

### 2.4. Embryonic vs. Adult HSCs

It has recently been shown that HSCs in the fetal liver divide using aerobic energy generation pathways, whereas adult HSCs from bone marrow use anaerobic pathways [85,86]. In general, the environment of embryonic progenitors of HSCs, as well as HSCs in the fetal liver and placenta, is oxygenated, which affects the metabolism and reproduction rate of these cells. In the embryonic precursors of HSCs, the mTOR pathway is activated, which provides an active metabolism of carbohydrates and fats [49]. This mTOR activation is highly dependent on thrombopoietin signaling [87]. Note that blocking the mTOR pathway prevents bone marrow adult HSCs division and preserves their quiescence [88].

Mature adult HSCs in the bone marrow, in contrast to embryonic dHSCs, have certain specific properties. Adult HSCs are dormant, have reduced metabolism, are predominantly anaerobic glycolytic, rarely leave the niche, and divide only rarely. Adult HSCs are characterized by heterogeneity, whereas fetal liver HSCs is homogeneously uniform in proliferation potential and differentiation [89]. Along with balanced multipotent cells, there is a pool of diverse HSCs that are biased to develop into a particular blood lineage [90,91]. Compared to embryonic HSCs, adults have a significantly reduced regenerative potential [92].

Embryonic HSCs tend to proliferate with insignificant loss of regenerative potential. Passing around eight divisions during the maturation and formation of the primary pool of HSC precursors, and then in the fetal liver, they retain the ability to self-renew and maintain the hierarchy of haematopoiesis. They retained a high lymphoid and myeloid differentiation potential [32].

It is noteworthy that the first clonogenic precursors with myeloid, megakaryoid, and erythroid potentials appear earlier than HSC precursors and form in embryogenesis as separate lines serving the immediate needs of the embryo, gradually moving from primitive to more definitive haematopoiesis [78,93,94,95,96]. Moreover, these erythro–myeloid progenitors (EMPs) appear first in the yolk sac and then in the dorsal aorta of the embryo at the beginning of the establishment of blood circulation [97,98]. It remains discussable whether these EMPs and early lymphoid progenitors originate de novo in the embryo or whether they spread from the yolk sac into the embryo [32,41,43,93,96,99,100,101,102,103].

Recent studies have shown that HSCs lose their ability to produce mast cell precursors during development in the fetal liver [104]. Adult HSC cells are also unable produce mast cells in steady-state haematopoiesis [105]. It has also been shown that macrophage populations of microglia, Kupffer cells in the fetal liver, and Langerhans cells in the skin are derived from EMPs from the yolk sac, independently of HSCs [106]. However, researchers demonstrated the colonization of microglia by macrophages and skin by mast cells derived from the bone marrow after tissue damage [107,108,109] Apparently, during development, the colonization of tissues by some types of myeloid cells occurs even in embryonic development, and they are able to exist there for a long time; however, the plasticity of HSCs will allow the restoration of tissue myeloid cells, ensuring their regeneration after injury.

These hematopoietic embryonic lineages persist independently of the pool of HSCs, in some tissues of the adult organism for almost their entire life [93]. These cells are self-sustaining, and their specific role in adult myeloid haematopoiesis during aging remains poorly understood and has been intensively studied in the last decade [22].

If we compare HSCs from an embryo with HSCs from adult organism, then one of the important differences is the efficiency of the DNA repair system. Mutations that cause various proliferative diseases are rare in young HSCs [110]. At the same time, young HSCs during division have a high activity of mTOR-dependent metabolism. Obviously, to compensate for possible damage from high metabolism and rapid division, HSCs in the fetal liver significantly increase the expression of DNA repair and antioxidant genes [85].

After migrating to the bone marrow, HSCs rarely divide during adulthood. According to various estimates, on average, dormant HSCs divide no more than 3–4 times during 2–3 years of mouse life [111]. However, despite rare divisions and a dormant state with a low metabolism, HSCs in the bone marrow age and accumulate mutations that change their differentiation potential and contribute to the exit from the dormant state. This determines both the predominance of myeloid shift and the formation of clonal haematopoiesis (CH). CH is when a limited number of HSCs that have accumulated mutations with a selective advantage determine high proliferative activity and production of a whole domain of mutant blood cells.

Although the embryonic precursors of HSCs in humans have not yet been identified, it is assumed that the picture of the embryonic development of HSCs and adult haematopoiesis in higher mammals proceeds according to a similar scenario (Figure 1).

## 3. HSCs Adulthood and Aging

Perhaps, aging of HSCs begins immediately after migration to the bone marrow and the most significant differences can be found by comparing the transcriptional differences of embryonic HSCs with the same cells of the mature and old organism.

When a pool of hematopoietic stem cells settles in the bone marrow, homing into the hypoxic niche, HSCs reduce the rate of division and slow down metabolism while retaining their main properties: the ability to self-renew and the ability to maintain complete haematopoiesis by differentiating into the precursors of all blood lines. Persisting in a dormant state, the HSC pool provides an emergency reserve for haematopoiesis, protected from environmental influences and the pressure of the mutation process. Some of the HSC enter asymmetric differentiation during severe injuries, blood loss, toxic damage, or infectious induced lymphopenia. Whereas during the ongoing steady-state adult haematopoiesis, HSCs division are very slow, and “on-duty” haematopoiesis is partially provided by numerous multipotent and unipotent specialized blood precursors [111,112].

With age, the pool of HSCs and multipotent precursors gradually increases its heterogeneity, acquiring a tendency to predominantly differentiate in one direction (e.g., myeloid, or lymphoid), whereas the pool of truly multipotent HSCs capable of maintaining long-term haematopoiesis decreases [113,114,115].

HSC aging is accompanied by significant changes in gene expression, their metabolism and physiology. However, to understand the exact causes and detailed interpretation of the mechanisms of HSC aging, the application of new methods in additional studies and a systematic approach are required. In this section, the authors summarize the current knowledge of the most important mechanisms of HSC aging, which include epigenetic, transcriptomic, proteomic, and metabolic changes in senescent HSCs, based on recent publications.

### 3.1. DNA Damage

Growing with age, HSC heterogeneity is primarily dependent on DNA mutational changes or epigenetic modifications. The most important cause of age-related changes in HSCs is the accumulation of mutations and DNA damage, especially in genes of the repair system [116]. This is indicated by the accumulation of phospho-H2AX (γ-H2AX), a marker of DNA double-strand breaks in elderly individuals, which is observed in aging HSCs in response to external and internal stress [117]. The role of DNA damage in aging HSCs was demonstrated in patients with Fanconi anaemia and in mice deficient in different DNA repair genes [118,119]. Mutations in genes involved in the DNA damage response can cause HSCs senescence, depleting them from the functional pool [120].

External and internal factors, such as replicative stress or high levels of ROS due to impaired mitochondrial activity, damage the DNA of hematopoietic stem cells during the cell cycle. Thus, the age-related damage to mitochondrial DNA (mtDNA) contributes significantly to the dysfunction and aging of HSCs [26]. Conversely, the A5178C mutation in the *Mt-nd2* gene, which encodes NADH dehydrogenase-2, results in a decrease in ROS and has been shown to protect mitochondria from oxidative damage and promote longevity [121]. Pharmacological enhancement of mitochondrial membrane potential in aged HSCs in vivo can rejuvenate HSCs, prevent DNA damage, increase engraftment potential in transplantation, and reverse myeloid bias in peripheral blood [122].

In addition, damage to mitochondria due to the accumulation of mutations in mitochondrial DNA lessen their functional activity. For example, by reducing the output of superoxide dismutase, which is involved in the defense against an oxidative DNA insult by ROS. A high level of ROS exacerbates DNA mutagenesis [123].

The inflammatory microenvironment may also have a negative effect on DNA damage in HSCs [124,125]. Recent work points to the important role of anti-inflammatory systems in maintaining DNA stability in HSCs. The expression of the NLRP12 (NOD-like receptor 12) is induced by DNA damage and recovers of the functional activity of HSCs in serial transplantations [126,127,128].

As noted above, the majority of adult HSCs to prevent exhaustion and damage persist in a dormant state only occasionally entering to division and differentiation to maintain haematopoiesis. Unexpectedly, DNA damage also accumulates in quiescent HSCs [13,126]; however, the rate of mutations in actively dividing HSCs is significantly higher [129].

Recent studies showed that HSCs/MPPs, on average, accumulate around 17 mutations and lose 30 base pairs in telomere regions per year starting at birth. So far, only 22% of all driver mutations that cause clonal proliferation have been identified, which demonstrates the polygenic nature of DNA damage in hematopoietic stem and progenitor cells [130].

### 3.2. Epigenetic Modifications

Epigenetic modifications in aging HSCs are characterized by a global change in DNA methylation status [131]. With age, both regulatory regions of genes associated with self-renewal and HSC differentiation undergo hypermethylation [132]. This hypermethylation contributes to the age-related increase in the number of HSCs while reducing their regenerative potential.

The relationship between DNA methylation status and the observed age-related changes in HSCs (skew towards myelopoiesis, defective, or enhanced self-renewal) were confirmed in experiments on changes in the activity of DNMT1 (DNA (cytosine-5-methyltransferase) and TET1 (10-11 translocation methylcytosine dioxygenase-1) methylation and demethylation agents, respectively. *Tet2*−/− HSCs exhibit significant changes in DNA methylation of transcription factor binding motifs that impair transcriptional priming for differentiation into several blood lineages [133]. Hypomethylation of DNA is often associated with various histone modifications that activate gene transcription [131].

The described epigenetic modifications are accompanied by irregular changes in the expression of genes involved in DNA repair, metabolism, intracellular signal transduction, mitochondrial homeostasis, cell cycle regulation, and differentiation [91]. This impacts the phenotype and negatively affects the function HSCs with age.

Knockdown of the novel epigenetic regulator of hematopoietic differentiation *Kat6b* in young LT-HSCs impairs myeloid cell differentiation and increases erythroid bias. The authors proposed KAT6B as a new target for improving age-related immune function [134]. The age-related epigenetic changes described above disrupt the expression of important transcription factors and increase the phenotypic heterogeneity of the HSC pool [135].

### 3.3. Transcriptomic Alteration

Single cell transplantation revealed the functional heterogeneity of individual adult HSCs [90]. Studies of differentiation potential of adult HSCs have proposed a new paradigm for maintaining the hematopoietic hierarchy through a pool of precommited HSCs [136,137]. Recent research in single cells transcriptomics confirmed and extended the findings of previous functional studies. Fifteen transcriptomic signatures were identified among the pool of HSCs, with some HSCs exhibiting linear differentiation into mastocytes, neutrophils, erythrocytes, or lymphoid-restricted lineage signatures. Changes in the differentiation potential of HSCs with age were also confirmed. Some HSCs show an epigenetic or mutation-driven transcriptomic switch in differentiation towards megakaryocytic-erythroid progenitors and cell cycle alteration. This causes an increase in susceptibility to myeloid haematopoiesis and an increased risk of neoplastic processes [138,139].

Significant heterogeneity of HSCs aging at the single cell transcriptome level has also been noted in recent studies. Based on 16 individual transcriptome datasets, poor reproducibility of differentially expressed genes between young and old HSCs was revealed.

This highlights the diversity of genetic mechanisms that implement age-related changes. Interestingly, about half of the differentially expressed genes revealed in different studies are cell membrane proteins/adhesion molecules (*Alcam, Cd34, Clca3a1, Dsg2, Itgb3*, and *Selp*,) and cytokine receptors (*Csf2rb, Ebi3, Flt3, Ghr, Il1rapl2*, and *Osmr*). Apparently, the role of these genes in the biology of HSCs was previously underestimated [91,140].

Accumulation of mutations, epigenetic modifications, and as result transcriptional changes negatively affects the physiology and function of HSCs and with age leads to disruption of various aspects of haematopoiesis reducing the protective function of the immune system. As result, the transcriptional and functional heterogeneity of HSCs increases with age.

### 3.4. Self-Renew of Aging HSC and Its Repopulation Ability

The age-related accumulation of mutational, epigenetic, and transcription changes awaken HSC from dormant state, and they enter into proliferation without physiological mitogenic signals. As a result, the proliferative activity and the number of malfunctioning cells with the HSC phenotype increase with age, but the ability of HSC to self-renew and to produce various blood lineages in a balanced way decreases [141,142].

Reciprocal transplantation of HSCs between young and old animals is used to answer the question about the endogenous and exogenous causes of aging of HSCs. Despite the higher proliferative activity of old HSCs, their repopulation efficiency for restoring the immune system decreased by 5 times, and their homing ability by 4 times [143]. Decreased ability to properly self-renew also reduces the pool of the most powerful HSCs. It is assumed that increased proliferation of aging HSCs is one of the main reasons for the tendency in elderly people for haematological diseases [4]. Clinical studies also showed that older HSCs have a decreased ability to repopulate and restore the immune system of recipients. Younger donors give less non-relapse mortality and better survival in recipients with haplotype-mismatched transplantation [144].

According to modern concepts, the balance between the quiescent and primed states of HSCs plays an important role in the ability to self-renew and repopulation of the hematopoietic system [145]. Some aging HSCs leave quiescence state, become metabolically active, and accelerate the cell cycle, eventually losing their stemness. Age-related increase in HSCs metabolic activity is also associated with a decline in transplantation potential. Recent work has shown that long-term treatment with a form of vitamin B3 (nicotinamide riboside, NR) enhances SIRT3 signaling and mitophagy, reboots juvenile metabolic potential, and restores the repopulation ability of HSC recipient mice [146].

Expression of *Tcf15* (transcription factor 15) indicates the most primitive subset of true multipotent HSCs. In addition, TCF15 is essential for maintaining HSC quiescence and long-term HSCs self-renewal [147]. Exit from the quiescence state is accompanied by downregulation of TCF15 and by an increase in intracellular aspartate levels, which leads to the proliferation of HSCs but to a lesser extent to differentiation to colony-forming progenitors [147,148]. It was revealed that aspartate in HSCs is used to produce asparagine and purines, which are necessary for the proliferation of HSCs [148].

BRCA1-deficient HSCs expand in the bone marrow of mice, culminating in functional exhaustion and failing to restore haematopoiesis in irradiated recipient mice [149].

In steady state HSC, activation chaperone-mediated autophagy is enhanced, which is necessary for protein quality control, fatty acid metabolism, and glycolysis [150]. Autophagy activity is reduced in aging HSCs, leading to impaired “self-purification”, to decrease in the number of functional HSCs and, consequently, to a reduction of repopulation capacity. It is assumed that autophagy is one of the mechanisms of HSC stemness maintenance. Furthermore, autophagy activity in aging granulocyte–monocyte progenitor (GMP) is not reduced, which may contribute to an increase in myelopoiesis with age (myeloid bias) [151,152].

### 3.5. Clonality and CHIP-Mutations

The efficiency of immune system cell replenishment and renewal plays an important role in maintaining its functions during life. It is well known that age-related changes in the HSC pool reduce regenerative potential, induce excessive proliferation, and provoke various immune disorders downstream, including cytopenia, primary myelopoiesis, and an increased risk of neoplastic processes [130].

Point mutations in genes that accumulate in HSPCs of healthy people with age and are transmitted in hematopoietic clones during ontogenesis are called CHIP mutations (Clonal Haematopoiesis of Indeterminate Potential). The genetic mutation cargo carried by each HSCs clone determines their transcriptional heterogeneity and also causes epigenetic and proteomic changes characteristic of aging immune cells [153]. Thus, the entire progeny of the original HSCs clone, has an identical set of somatic mutations and modification. Steady-state, haematopoiesis is maintained by many HSCs and multipotent progenitors; however, during aging, the selective advantage of some HSCs provides advantage of some hematopoietic clones originated from small number of HSCs with a unique mutational profile [154]. With aging, more and more blood cells originate from fewer and fewer HSCs. The haematopoiesis of people under 65 is predominantly provided by a high diversity of haematopoietic stem and progenitor cells (HSPCs) including HSCs/MPPs—about 20,000–200,000. However, after 70, around 30–60% of blood cells come from 12–18 HSC clones, whereas each of clone can occupy 1–34% of the total haematopoiesis [130].

Aging of HSCs is one of the causes of haematopoiesis clonality. With age, as a result of an asynchronization and disruption of the DNA repair system and an increase in age-dependent inflammation, some HSCs lose their function because of accumulation mutation load or inflammatory exhaustion, and finally entering a state of senescence [155]. While the other part, under the influence of inflammatory stress and external and internal stimuli, begins to actively proliferate, which increases the risk of driver mutations accumulation that contribute to the selective advantage of some HSCs. The symmetrical propagation of HSC clones or the advantage in the asymmetric differentiation of one or more HSCs generates haematopoiesis clonality [17,130,156].

**Figure 2:**
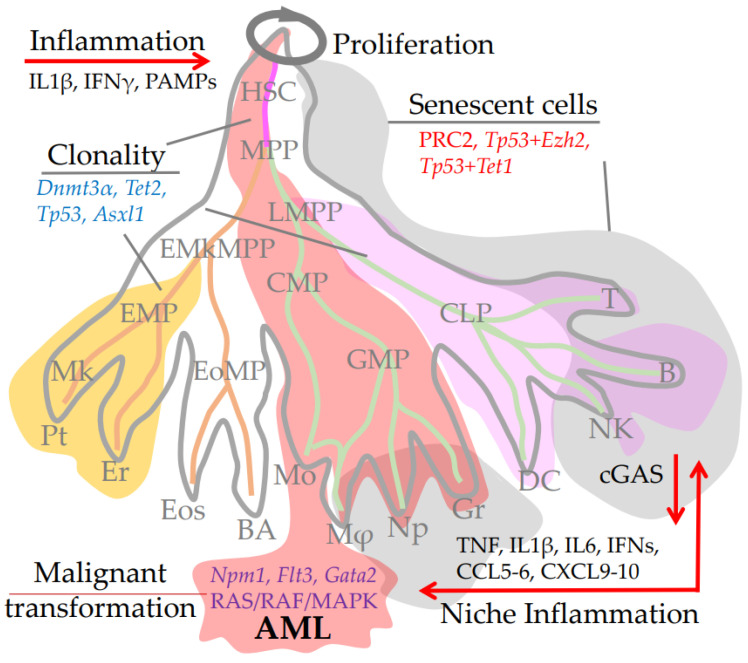
Consequences of aging in the adult hematopoietic hierarchy: accumulation of senescent cells, systemic inflammation, hematopoietic clonality, and proliferative diseases. Examples of three clonal hierarchies are highlighted with a megakariod–erythroid (yellow), a myeloid (red), and a lymphoid bias (pink). Note, the hierarchy can expand and capture all branches of haematopoiesis, depending on the level of stemness of the mutant precursor and on the type of mutation. The blue colour font at the top shows mutant genes that cause proliferation (grey circle) and/or clonal differentiation of HSPCs. Inflammatory factors that accelerate these processes are shown in black font for systemic infectious inflammation (**top left**) and niche inflammatory factors (**bottom right**). The direction of influence of these factors on HSC/HPC aging, malignant transformation, and the formation of a pool of senescent cells (grey area) is shown by red arrows. Mutations causing hematopoietic clonality are shown in blue font (**upper left**). Double mutations that accelerate cellular senescence are highlighted in red font (**top right**). An example of malignant transformation from myeloid clonality to acute myeloid leukaemia (AML) due to additional mutations or signaling pathway activation (dark blue font, **bottom**) is shown at the bottom of the figure.

CHIP mutations disrupting TP53 (tumour protein p53), which drives clonal haematopoiesis, have been identified in humans and mice [157,158]. Mechanistically, the p53 mutation promotes the clonal expansion of HSPCs by enhancing the expression of the *Ezh2* gene which leads to the activation of self-renewal and differentiation genes. Inhibition of EZH2 reduces the repopulation potential of HSPC clones carrying p53 [157]. Additional mutations in the PRC2 (The methyltransferase Polycomb Repressive Complex 2) Destabilizing PRC2-complex which consists of EZH1, EZH2, SUZ12, and EED subunits, cause cell cycle arrest that overcomes the induction of p53 clonality, and cells exhibit senescence-like phenotypes [159,160,161]. In addition, the loss-of-function double mutation of *Tp53* and *Tet1* (10-11-translocation-1) also induces senescence [162], illustrating subtle genetic regulation between apoptosis, senescence induction, and clonal proliferation (see Figure 2).

Clonal haematopoiesis increases risk of malignant blood transformations, age-related diseases, including cardiovascular disease, cancer, and the risk of all-cause mortality [18,156,158,163]. Recent studies identify specific CHIP-mutations that cause clonal haematopoiesis and, consequently, a high risk of malignant blood transformations. The most cases of clonal haematopoiesis are associated with mutations in only three genes: *Dnmt3α*a (DNA methyltransferase 3 alpha), *Tet2* (10-11-translocation-2), and *Asxl1* (Additional Sex Combs Like 1, Transcriptional Regulator) [164]. In another study, the most common non-cancerous hematopoietic clonal mutations were found in *Dnmt3α*, *Tet2, Tp53*, and *Runx1* (Runt-related transcription factor 1) [165] (Figure 2). These genes regulate cell division and differentiation at both the transcriptional and epigenetic level. However, mutations in other genes also appear to be required for the onset of clonal haematopoiesis followed by malignant transformation.

Mutation in both *Dnmt3α* and *Npm1* (nucleophosmin 1) causes clonal haematopoiesis followed by myeloproliferative disorder and successively acute myeloid leukaemia (AML), (Figure 2). The transition from clonal haematopoiesis to myeloproliferative disorder is accompanied by the selection of mutations that activate RAS/RAF/MAPK signaling [166].

The mutation in *Dnmt3α* is often followed by mutations in *Jak2* (Janus Kinase 2) and spliceosomal genes *Sf3β1* and *Srsf2*. The number of clonal haematopoiesis cases associated with mutations in *Dnmt3α* and *Jak2* increases linearly with age, and starting from the age of 70, those associated with additional mutations in *Sf3β1* and *Srsf2* (splicing factor 3β1 subunits and serine and arginine rich splicing factor 2) grows exponentially [167].

Mutations in *Tet2* and *Flt3-*ITD (Fms Related Receptor Tyrosine Kinase 3—internal tandem duplication) in mice result in fully penetrant lethal acute myeloid leukaemia followed by extensive remodeling of DNA methylation. Overexpression of *Gata2* reversed AML stem cell differentiation and attenuated leukemogenesis, demonstrating at least some dependence of such transformation on GATA2. These studies once again confirm the role of hematopoietic clonality in malignant transformation (Figure 2) [168]. Note, FLT3-ITD translocation in the FLT3 receptor constitutively activates downstream signaling pathways leading to uncontrolled proliferation of immature leukocytes. Further analysis of these mice showed significant deregulation of factors involved in megakaryocyte development and platelet production, including *Gata1, Zpfm1, Rac1*, and *Ehd2*. Thus, placing double *Tet2* and *Flt3*-ITD mutated cells into a rigid framework of myeloid differentiation and proliferation [169].

Thus, clonality is the result of several processes associated with aging: the accumulation of driver mutations that promote selective proliferation, epigenetic changes conferring selective advantages on the differentiation and/or proliferation of individual HSC, or HSPC further stimulated by age-dependent niche or autocrine inflammation (see Section 3.7) [17,170].

As a result, individual HSC clones invade haematopoiesis, narrow the cellular repertoire, leading to anaemia and immunodeficiencies. The proliferation and accumulation of individual hematopoietic clones increases the risk of cardiovascular disease and by accumulation of additional carcinogenic mutations leading to proliferative blood diseases, affecting health and reducing life expectancy [18].

### 3.6. Differentiation Imbalance, CHIP, and Chromosomal Alterations

The majority of CHIP mutations (~87%) determine the selective advantage of myeloid lineage differentiation, and only the remaining 13% of mutations control lymphoid differentiation [110,163,171]. Mutations with gain or loss of function in key genes responsible for HSC commitment determine the differentiation of a particular clone in a predominantly one direction (e.g., lymphoid, megakariod, or myeloid). It is assumed that this occurs due to intrinsic defects and a certain mutational profile of old HSCs [130]. Perhaps, different mutation rates in different genes contribute to the accumulation of mutations in the sets of genes that determine the susceptibility of HSCs to myelopoiesis or interfere with lymphopoiesis. Most abundant *Tet2* mutation in HSPCs in aged mice and humans causes proliferation and myeloid bias [172].

Mutations associated with myeloid haematopoiesis accumulate faster in the elderly population than mutations associated with lymphoid bias [173].

However, some specific genetic events may contribute to lymphoid clonal bias. Mosaic chromosomal alterations, which are mostly associated with lymphoid malignancies and provide a predominance of lymphoid haematopoiesis [110,174], can appear with age in healthy individuals.

The transcription factor EBF1 activates the HSC differentiation program towards B cell lineages. Distortion of EBF1 transcription factor expression cause strong myeloid bias. Because EBF1 represses myeloid differentiation factor C/EBPα, a mutation in EBF1 increases C/EBPα expression and promotes myeloid differentiation in HSCs and multipotent blood progenitors [175]. Conditional knockout of *Ebf1* converted HSC into innate lymphoid cells (ILCs) and T cells [176]. A *Gata2* mutation in one of the DNA strands results in haploinsufficiency, which also causes a myeloid bias in HSPCs [177].

Mitochondrial activity also contributes to myeloid differentiation. A disturbance of mitochondrial metabolism by *Atm* knockout (Ataxia Telangiectasia Mutated Serine/Threonine Kinase) inclines HSCs to myeloid differentiation, and when this metabolism is normalized, HSCs restore the lymphoid potential [122,178]. It is assumed that the differentiation of lymphocytes requires more energy costs compared to myelopoiesis.

As recently documented, megakaryocyte/platelet developmental bias also related to an increase in the number of HSCs that are prone to differentiation into megakaryocytes during aging [179]. Comparison of HSCs obtained from old and young mice at the single cell level showed that aging correlated with increased expression of platelet-specific genes. The transplantation study shows that many old HSCs are prone to megakaryocytic differentiation, which explains the age-related increase in platelet count in mice. Meanwhile, the expression of *Zfpm1/Fog1* (Zinc Finger Protein, FOG Family Member 1) the regulator of erythroid and megakaryocyte cell differentiation increases significantly with age. Knockout of *Zfpm1/Fog1* gene completely restores the lymphoid potential of aging HSCs measured by mice transplantation [180].

The dynamic balance between the heterogeneous pool of long-term and short-repopulating HSCs changes with age. It was shown that long-term and short-term HSCs give rise to predominantly myeloid and lymphoid lineages, respectively. And a change in the ratio of these cells with age can lead to a myeloid skew [181]. All of these data suggest that myelopoiesis occurs by default, whereas several additional external and internal signals are required for lymphopoiesis [182,183,184]. Probably, lymphopoiesis has a more complex differentiation program, since it is evolutionarily younger [185]. Therefore, we hypothesized that the age-related accumulation of mutations in HSCs would rather prevent the implementation of a more complex multigene program of lymphoid differentiation, and thus the default, evolutionary older program of myelopoiesis is more likely to obtain a selective advantage.

### 3.7. Inflammation and HSC Niche Aging

Increasing inflammation with age inflammAging (iAging) impairs all aspects of health associated with age. Age-related inflammation occurs for many reasons, including the accumulation of mutations in inflammation-suppressor genes, the spreading of chronic infections, and the accumulation of senescent cells that secrete inflammatory cytokines [186].

The studies on mice showed that inflammation enhances with age due to certain epigenetic and transcriptomic changes, as well as the activation of immune response signaling pathways by various reason [187].

An increase in the number of senescent cells with a Senescence-Associated Secretory Phenotype (SASP) occurs with aging in the body, which contributes significantly to iAging and, ultimately, aging of the bone marrow niche and HSCs [188].

The bone marrow niche aging causing aseptic chronic inflammation and secretion of pro-inflammatory cytokines (TNF, types-I and -II interferons) cause HSCs aging [189,190]. Moreover, recurrent, or chronic inflammation can impose HSCs imprinting, which ultimately affects the differentiation potential and can cause various disorders of haematopoiesis.

Under the inflammation, HSCs are activated from a quiescence state, proceed to proliferation or differentiate into more mature immune cells. In the process of proliferation and differentiation, HSCs gradually lose their ability to self-renewal. Inflammation can cause exhaustion/senescence of HSCs and increase the risk of malignant transformation [19]. Even after acute inflammation, the functional pool of HSCs can be significantly depleted and show no signs of recovery within a year [191].

There is significant evidence that bacterial and viral PAMPs (pathogen-associated molecular patterns), directly affecting niche and HSCs via its PRR (pattern recognition receptors). Such signals awaken HSCs from their dormant state [192]. Apparently, with persistent inflammation, HSCs occurs an imprinted epigenetic rearrangement (an analogue of trained immunity), which determines the predisposition of HSCs to myeloid bias [193].

The inflammation also intensifies myelopoiesis. After transplantation of young HSCs to old animals, myeloid haematopoiesis prevails. Somewhat unexpectedly, myelopoiesis also predominates after transplanting old HSCs to young animals [116,194]. Note, the radiation to which the animals are exposed before transplantation itself induces an inflammatory response. In experiments where chemotherapy with busulfan, which does not cause inflammation, was administered before transplantation, no myeloid skew was observed [195,196]. This emphasizes the importance of the inflammatory background for the myeloid bias [197,198].

The gut microbiome can contribute iAging. Inflationary microbial PAMPs enter the blood through the damaged wall of the gastrointestinal tract, most often because of local inflammation. PAMPs stimulate an increase in IL-1α/β expressions in the bone marrow by binding with TLR4 and TLR8. HSCs are also damaged by this or a similar inflammatory mechanism. Moreover, hematopoietic stem cells in germ-free or IL-1R1 knockout mice retain their repopulation potential as shown in transplant experiments. Blocking IL-1 or suppressing the microbiome with antibiotics leads to similar HSCs antiaging effects [199].

Niche inflammation plays a role of fuel in clonal haematopoiesis. As mentioned above, clonal haematopoiesis is associated with mutations in the *Tet2* gene. However, successful propagation of mutant clones is dependent on IL-1 signaling, highlighting the role of inflammation in the development of clonal haematopoiesis [200]. Moreover, HSCs bearing *Tet2* gene mutation are sensitive to inflammatory stimuli. Inflammation of the gut caused HSPCs to proliferate and put them into a pre-leukemic state. After exposure to antibiotics in *Tet2*−/− mice, the pre-leukemic state of HSPC was prevented [201].

Chronic mycobacterial infection induces an IFNγ-mediated inflammatory response and stimulates the proliferation of hematopoietic stem and blood progenitors carrying the *Dnmt3α*−/− mutation but does not affect those without the mutation [202].

Moreover, mutation of the *Tet2* gene in HSCs induces the secretion of pro-inflammatory cytokines in downstream progeny, which can accelerate the aging of the surrounding niche and, by an autocrine mechanism, induce senescence and enhance the clonal expansion of HSCs [203]. TET2 is also repressor of inflammatory program in myeloid cells. Mutation in *Tet2* upregulates several inflammatory cytokines in myeloid cells including that specific for SASP senescent phenotype (e.g., IL-1β, IL-6) [204,205]

Thus, mutations in the *Tet2* gene, either in HSCs or niche cells, stimulate proliferation and an increase in the inflammatory response, typically leading to inflammation-induced senescence and ultimately, to the selection of dominant hematopoietic clones (Figure 2).

Bone marrow niche and microenvironment play an important role in the physiology and maintenance of the HSCs pool. The senescent BM stromal cells and endothelial cells produce IL1β, IL6, and other inflammatory factors such as CCL5, CCL6 CXCL9, and CXCL10, which are involved in HSC aging [206]. Moreover, the different composition of mesenchymal cells of the bone marrow niche secreting various inflammatory cytokines significantly determines the myeloid polarization of HSCs. Enrichment of the niche with specific TNFα-secreting inflammatory mesenchymal cells triggers the TNFα/ERK/ETS1 pathway, which increases IL27Ra expression on the surface of HSCs. Activation of this receptor inhibits ability of HSCs to self-renew, impairs long-term hematopoietic reconstitution, and deteriorates both myeloid and lymphoid differentiation after transplantation [207].

Several niche molecules involved in various aspects of the HSC life cycle and function are affected during niche aging and inflammation, including VCAM1, CXCL12, SCF, and G-CSF [208]. It has recently been demonstrated that Neogenin-1 is specifically expressed in the most quiescent HSCs. The interaction neogenin-1 on HSCs with netrin-1 on endothelial or periarteriolar stromal cells maintains the quiescence state, and loss of niche expression of netrin-1 with age leads to excessive proliferation of HSCs [209]. In line, netrin-1 has also been shown keeping cells from excessive inflammatory response [210].

Macrophage dysfunction in the aging niche, via the IL1B signaling pathway, forces HSCs to differentiate towards megakaryocytes/platelets. In young mice, depletion of phagocytic cells or disruption of *Axl*-receptor tyrosine kinase (the receptor for efferocytosis involved in the removal of apoptotic cells) expanded the pool of HSCs prone to differentiate into megakaryocytes [211].

Reciprocal HSC transplantation experiments demonstrate that the young niche largely restored the transcriptional profile of aged HSCs, but not their DNA methylation profiles. These experiments determine the limited ability of the young niche to restore aged HSCs function [212]. RhoGTPase (*Cdc42*) is a mediator of chronic inflammation, inflammasome assembly, and *Cdc42* overexpression enhances cell division in HSCs [213]. These events lead to loss of repopulation potential, to myeloid bias, and other signs of HSC aging. Treatment of old HSCs with a *Cdc42* inhibitor called CASIN and transplantation into a young niche rejuvenated HSCs. The authors also remind that niche aging is partly due to low levels of osteopontin in the old niche. Thus, HSC rejuvenation requires a youthful niche and control of the internal mechanisms of HSC aging [214].

## 4. HSCs Senility and Immune Aging as a Risk Factor for Pathologies

During ontogenesis, HSCs support haematopoiesis and give rise to all specialized cells of the immune system. However, the aging of differentiated cells of the immune system itself contributes to the aging of the whole organism in addition to the aging of the HSC pool. The immune system provides a defense against infections and cancer, maintains tolerance to autoantigens, and participates in tissue homeostasis by supporting regeneration and removing senescent and transformed cells. However, with age, the functional activity of immunity declines, and the protective capacity of the immune system decreases. In this section, the authors describe the major milestones and mechanisms of the immune system aging, focusing on the role of HSCs in this process.

### 4.1. Lymphopenia, Anaemia, Thrombosis, and Aging

Lymphopenia makes a significant negative contribution to the aging immune system. Various stresses, physical, chemical, or biological factors that the body encounters during life can induce lymphopenia, which triggers homeostatic proliferation, a physiological process of numerical restoration of the peripheral pool of T-lymphocytes after lymphopenia [215,216,217]. As has been shown in the context of the COVID-19 epidemic, lymphopenia can be a consequence of the infectious process, and is also a predictor of the severity of the disease and complications [218]. Depending on the severity of lymphopenia, this process may acquire pathological features and contribute to the selection of potentially autoreactive clones of T-lymphocytes [215,216]. The effectiveness of this recovery process depends on the age characteristics of the regenerative potential of the hematopoietic system [191,219].

Lymphopenia of various origins can lead to incomplete removal of the infection and the transition of this process to the chronic phase. Chronic infections raise the inflammatory level and, together with lymphopenia, give excessive signals to bring a significant pool of HSCs to proliferation to restore haematopoiesis, which further depleting HSCs in the elderly. Such events contribute to complications from severe infections and organism frailty.

Anaemia, another aging complication, occurs in about 18% of older people over 80 years of age [220]. Anaemia can be caused by nutritional factors, blood loss, chronic kidney disease, chronic inflammation, or clonal haematopoiesis [221,222]. The latter two factors are directly related to aging processes in the hematopoietic system [223,224]. The exact mechanisms of clonal haematopoiesis-related anaemia are unknown. However, a mutational signature associated with anaemia in the elderly in clonal haematopoiesis was identified [225]. In addition, driver mutations were shown to accelerate clonal haematopoiesis and increase the rate of acquisition of additional mutations, which may adversely affect erythropoiesis [226].

In the elderly, an increase in platelets count has been observed in normal healthy aging in both humans and primates [227,228]. Due to the accumulation of platelets and other pathologies associated with vascular disorders, risk of thrombosis increases dramatically in the elderly [229].

As noted above, aging HSCs lose their normal regenerative potential. The megakaryocytic progenitor also undergoes changes; the aging is associated with an increase in the number of platelets. With age, thrombopoiesis maintained by accumulating immediate megakaryocyte progenitors (MkPs). Indeed, as shown in functional tests on mice, MkPs had a much greater regenerative potential than their counterparts from young mice, despite the fact that HSCs from older mice could not give a sufficient repopulation of platelets after transplantation [230].

As described above, the chronic inflammatory background inflammatory aging characteristic of aging can also contribute to a shift from the optimal number of megakaryocytes, which increases the risk of thrombosis, age-related arrhythmia, and ischemic stroke [231,232].

Anaemia or microthrombosis introduces the body to the stress of pressure surges, which through the renin–angiotensin system (RAS) affects not only blood pressure but also haematopoiesis. RAS signaling pathways have been shown to be able to trigger the proliferation of cord blood HSPCs [233]. Moreover, RAS receptors, including angiotensin-I converting enzyme (ACE/CD143), were found on the HSCs surface from early embryo to adult [50,234].

### 4.2. Loss of Naïve T-Lymphocytes and TCR Repertoire

A steady decline in the production of naïve T- and B-lymphocytes due to involution of the thymus and decreased function of both primary and secondary lymphoid organs including bone marrow decline, resistance to infections, and increase risk of cancer [11]. Such age-related decreases in the influx of naive lymphocytes leads to a suboptimal course of age-related lymphopenia and exacerbates the negative consequences of homeostatic proliferation [215,235]. In general, this results in altered clonal organization and decreased protective diversity of antigen–recognition receptor repertoires [10,236,237]. Thymic involution is thought to result from a decrease in the number of early precursor T-cells that populate the thymus [238] However, an important role in the involution of the thymus is played by age-related depletion of the population of epithelial thymic precursor cells that form the cortical and medullary epithelial network [11,239]. Apparently, aging medullary thymic epithelial cells are unable to provide the proper level of presentation of their own antigens [10,11]. This impairs the efficiency of negative selection and leads to a narrowing of the T-cell receptor repertoire of T-regulatory cells produced by the aging thymus.

Impaired lymphoid precursors production decrease production of new B-cells by the bone marrow and T-cells by the thymus, as well as to a narrowing of the diversity of B- and T-cell receptors. At the same time, age-related changes in the thymus are accompanied by qualitative changes in the repertoires of newly formed effector and regulatory T-lymphocytes, which can negatively affect the pool of B-lymphocytes [240,241].

The concomitant accumulation of induced T-regulatory cells in the periphery also contributes significantly to the changes in the repertoire of antigen-recognition receptors in naïve lymphocyte populations [242,243]. A decrease in naïve lymphopoiesis, qualitative changes in thymopoiesis, and accumulation of peripheral changes in adaptive immunity with aging have a negative effect on the maintenance of autotolerance and efficiency of infectious and anti-tumour immunity including control of hematopoietic clones spreading [243,244].

### 4.3. Reduced Effectiveness of Antigen Presentation

Age-related changes in the HSC pool also negatively affect the innate immune system. The genetic load of aging HSCs is transferred to daughter cells of myeloid lines, disrupting their functional activity [245]. Despite an increase in the total number of myeloid cells with age, there is a lessening of their functions. The efficiency of interferon secretion, antigen processing, and presentation decreases with age. These age-related changes in the landscape of antigen presentation skew the balance between different subpopulations of lymphocytes [186,241,246,247,248]. The consequence of this is a decrease in ability of immunity to recognize “self”, “non-self”, and “altered-self”, as well as a decrease in the amplitude of the immune response [249]. The decrease in antigen presentation and subsequent decline in antigen processing impairs the ability of the immune system to recognize and destroy senescent cells in the body. This decrease in basic immune function, combined with a lessening in scavenger cell phagocytosis, significantly augments the amount of cellular debris and the number of senescent cells with age [250]. In several studies, a significant decrease in phagocytic activity associated with age was discussed [251,252]. It also shows the contribution of phagocytic activity to the pathogenesis of diseases associated with age [253].

Thus, a decrease in the process of antigen presentation does not allow immune surveillance to recognize infection, mutated clonal and malignant cells with sufficient efficiency. Decreased recognition and removal of remnants of apoptotic and senescent cells leads to their accumulation. Secretion of a set of pro-inflammatory factors by senescent cells increases the inflammatory background, which disrupts remodeling, tissue regeneration, and physiological functions of the whole organism [186,247,248]. This significantly reduces the protective properties of innate immunity.

### 4.4. Inflammatory Phenotype of Differentiated Immune Cells, Senescence and Aging

Proliferative exhaustion, mitochondrial dysfunction, oxidative stress, telomere shortening, chromatin disruption, epigenetic dysregulation, oncogenes activation, DNA damage, accumulation of mutations, and inflammation increase senescent cells number in the immune system [153]. Senescence is a protective mechanism that prevents unauthorized proliferation or differentiation in the immune system. The state of senescence is triggered by sensory mechanisms of damage to DNA or cellular stress and accompanied by epigenetic, transcriptomic, and proteomic alterations in the cell [187,244,254,255]. One such cytosolic DNA sensor that triggers the aging program is cGMP-AMP synthase (cGAS), which activates innate immunity in response to viral damage to cells. After a conditional knockout of cGAS in response to DNA damage, cells not able to turn on senescence and expression of inflammatory cytokines. Modulation of cGAS activity has been proposed as a new strategy for the treatment of senescent-associated diseases (cancer, neurodegenerative, and cardiovascular diseases) and aging [256]. The launch of the senescence program in response to DNA damage is accompanied by disruption of autophagy processes, dysfunction of mitochondria and lysosomes, activation of inflammasomes, and metabolic stress. As a result, there is a constant spontaneous production of SASP inflammatory factors released into the surrounding tissues [257,258,259,260,261,262]. The most important mediators of such systemic inflammation are IL-1β, IL-6, CXCL9, and TNF; their levels increase with age which is a main factor of iAging (Figure 2) [153,263,264].

Chronic inflammation causes cellular stress and proliferation, which increases the risk of autoimmune and cancerous processes. It is considered the cause of several age-related pathologies, such as atherosclerosis, hypertension, type 2 diabetes mellites, osteoarthritis, and also contributes to the development of some age-related neurodegenerative diseases [265]. Moreover, both adaptive and innate immune cells with senescent phenotype contribute significantly to the maintenance of the general inflammatory background in the body, which is typical for aging [266]. Aging is associated with an active infiltration of aged tissues (especially visceral adipose tissue) by immune cells. It was suggested that adipocytes and adipose tissue-associated macrophages may play a central role in the maintenance of chronic inflammation in old age [267,268]. Tissue inflammation activates remodeling and promotes fibrotic changes [269,270]. A senescent cell bystander effect induces a DNA damage response, in neighboring cells inducing further accumulation of senescent cells, which impairs various organs and tissues functional activity [229,271].

Thus, age-dependent accumulation of senescent cells forms a chronic inflammatory background, which, on the one hand, exhausts the immune system, causing stress and background proliferation, and on the other hand, senescent cells, accumulate in the bloodstream, losing their main immune function and weaken the immune system. In addition, due to the bystander effect, senescent cells stress and transform normal body cells (including the HSC pool) into senescent or pre-senescent ones. A “vicious cycle” ensues where the aging of the immune system exacerbates the aging of the body and pool of HSCs.

## 5. Conclusion Remarks

The current global demographic landscape is characterized by progressive population aging that increases the burden on healthcare systems. The number of people suffering from age-related diseases is increasing significantly. Age increases the risk of diabetes, atherosclerosis, osteoporosis, coronary heart disease, chronic cerebral ischemia, liver fibrosis and cirrhosis, autoimmune diseases, cancer, and chronic infections. The hematopoietic system is involved both in the prevention of senile diseases and in pathogenesis in case of its imbalance and aging. HSCs underlies the entire hierarchy of the hematopoietic system in humans and animals. Modern studies confirm the close relationship between HSC aging and a decrease in the functional activity of the immune system and an increased risk of developing age-related pathologies.

In this review, we traced the ontogeny of HSCs from their birth to old age. Embryonic and adult HSCs showed significant differences in their properties during ontogeny. Embryonic HSCs are characterized by a high level of proliferation, with high energy consumption, activation of antioxidant, and reparative signaling pathways. Embryonic HSCs have a high regenerative potential, the ability to repopulate all blood lineages, including mast cells, and produce numerous symmetrically dividing offspring that retain their stem properties.

In contrast, adult HSCs reside in a dormant state in a hypoxic bone marrow niche that protects them from environmental stresses. Adult HSCs are characterized by a low level of energy metabolism, a predominance of glycolysis, and increasing with age heterogeneity of differentiation and regenerative potential as well as a partial loss of capacity to produce mast cells, glial cells, and Langerhans cells under steady-state conditions.

Despite the high level of proliferation during embryonic development, which in terms of the number of cell cycles is comparable to the entire life of adult HSCs, embryonic HSCs do not accumulate mutational load. Adult HSCs, despite their rare proliferation during a lifetime, accumulate CHIP-mutations that increase the risk of haematopoiesis clonality. In the elderly, individual clones can occupy a significant part of haematopoiesis, which, on the one hand, increases the risk of malignant transformation, and, on the other hand, increases the number of malfunctional, stressed cells, which also results in the accumulation of senescent cells. Age-related inflammatory signals and/or driver mutations bring HSCs out of their dormant state, pushing them into proliferation. Thus, initiating the expansion of the pool of HSCs that have lost their stem properties, which reduces the number of pluripotent, transplantable HSCs in the bone marrow. The paradoxical contradiction of the influence of inflammatory factors on the development and aging of HSCs was noted in our review. Macrophages with an inflammatory signature and interferon signaling promote the development and proliferation of embryonic HSCs and their precursors, whereas in the adult niche, inflammatory macrophages and interferon signaling induce aging of HSCs.

Literature data have shown that myeloid bias characteristic of aging is a consequence of multiple aging processes, including the accumulation of CHIP-mutations, epigenetic modifications, disturbances in the transcription of differentiation factors, mitochondrial dysfunction, metabolic disorders, clonality, and chronic inflammation. An evolutionarily younger, more complex, and at the same time more energy-consuming mechanism of immunity is adaptive immunity. Age-related transcriptional desynchronization and disturbance of HSC metabolism causes disruption primarily in lymphoid differentiation. Similarly, random mutations are more likely to damage the more complex regulation of a lymphoid differentiation program.

Aging and loss of bone marrow cellularity reduce the release of lymphoid precursors to the periphery. In addition, involution and dysfunction of the thymus reduces the number of naive lymphocytes in blood circulation. At the same time, environmental stresses, including infectious lesions, deplete the adaptive immune system, which causes its aging and the accumulation of senescent populations of differentiated immune cells. Decreased influx of myeloid precursors from the bone marrow and accelerated aging of differentiated myeloid cells reduces the number of phagocytes and antigen presenting cells, as well as cells capable of responding to PAMP and pro-inflammatory stimuli. Accumulation of senescent cells with SASP increases the overall inflammatory background due to the constitutive secretion of several pro-inflammatory cytokines. Both systemic age-dependent inflammation and the accumulation of senescent macrophages and stromal cells in the bone marrow niche accelerates the aging of the HSC pool and is a negative background provoking excessive HSC proliferation, loss of pluripotency, and depletion of the immune system with an increased risk of lymphopenia, anaemia, and immunodeficiency.

Despite extensive research in this area, the exact reason and mechanisms of age-related changes in the hematopoietic system remain unclear. Comparative studies of the genetics and physiology of young HSCs will improve our understanding of the mechanisms of development of the hematopoietic system in the early embryo and will suggest possible ways of HSC pool regeneration. The study of the differences between the embryonic and adult niche brings us closer to understanding the processes of self-renewal without exhaustion, an increase in the mutational background, and the mechanisms for maintaining the differentiation potential and clonal diversity.

Currently, prospective studies of senolytic drugs are underway, and methods are being developed to slow down the aging process and prolong active longevity. However, effective therapeutic approaches that would meet the requirements of clinical safety have not yet been developed. Perhaps, a new trend focusing on the fight against clonal adaptive immunity will lead to the development of novel approaches to the treatment of age-related pathologies. However, it is necessary to further study the mechanisms of aging and search for therapeutic targets to prolong the active period of life and maintain healthy aging. In this context, the research in the field of embryonic haematopoiesis, aging HSCs, and aging branches of the adaptive and innate immune system seems to be relevant, since immunity is actively involved in the elimination of the aging and altered cells of the body and, apparently, can play a key role in the development of anti-aging therapeutic approaches.

## Data Availability

Where no new data were created.
